# International Committee on Systematics of Prokaryotes: minutes of the open plenary meeting, Tuesday, 22 October 2024, Sesto Fiorentino, Florence, Italy, and via Teams

**DOI:** 10.1099/ijsem.0.006621

**Published:** 2025-01-03

**Authors:** Aharon Oren

**Affiliations:** 1 The Institute of Life Sciences, The Hebrew University of Jerusalem, The Edmond J. Safra Campus, 9190401 Jerusalem, Israel

**Keywords:** ICSP, International Committee on Systematics of Prokaryotes, minutes, plenary meeting

## Abstract

A hybrid in-person and online open plenary meeting of the International Committee on Systematics of Prokaryotes (ICSP) was held on 22 October 2024 at the Consiglio Nazionale delle Ricerche, Sesto Fiorentino, Florence, Italy, and via Teams just prior to the IUMS 2024 Congress. To comply with Articles 4(d) and 5(d) (1) of the statutes of the ICSP, the minutes of this meeting are published here.

An open plenary meeting of the International Committee on Systematics of Prokaryotes (ICSP) was held on 22 October 2024 at the Consiglio Nazionale delle Ricerche in Sesto Fiorentino, Florence, Italy, in conjunction with the IUMS 2024 Congress. The meeting was also accessible online via Teams. According to Article 4(d) of the statutes of the ICSP[1], minutes of the plenary meetings of the ICSP are reported by publication in the *International Journal of Systematic and Evolutionary Microbiology* (*IJSEM*). According to Article 5(d) (1), it is the task of the Executive Secretary of the ICSP to submit the minutes of the plenary meetings for publication in the journal. To comply with the statutes, the minutes of this meeting are presented below.

## Minute 1. Welcome

S. Ventura, Research Director of CNR, Research Institute on Terrestrial Ecosystems, Sesto Fiorentino, Italy, and ICSP representative of the Italian Society for General Microbiology and Microbial Biotechnology (SIMGBM), hosted the meeting and welcomed the participants.

## Minute 2. Call to order

ICSP Chair E.R.B. Moore called the meeting to order at 14.00 CEST.

## Minute 3. Record of attendance

The meeting was attended in person by E.R.B. Moore (ICSP chair), P. Nielsen (ICSP treasurer), S.L.W. On (Secretary of Subcommittees; co-opted member), M. Göker [Secretary, Judicial Commission (JC)], ICSP full members M. Kostovski, J.-S. Lee and S. Ventura (member of the JC), ICSP co-opted members M. Hamada and M. del C. Montero-Calasanz (member of the JC) and guests I. Calegari Moia, J.-S. Kim and K.-I. Suzuki. The meeting was attended online by A. Oren (ICSP Secretary); H. Christensen (co-opted member; vice-chair, JC); ICSP full members M. Birbir, B. Duim, D. Hatzinikolaou, L.A. Maldonado Manjarrez, M. Seruga Musić, I.C. Sutcliffe, G.Y.A. Tan and A. Táncsics; co-opted members R.R. de la Haba, T. Eisenberg, M. Figge, M.-S. Li, A. Sidarenka and A. Thamchaipenet; and guests T. Ahmad, H.L. Banciu, D. Daffonchio, G. Gasparich, A. Guerrero Mendoza, A. Irsyadi A, M. Khalid, C.-H. Kuo, R. Marasco, O. Prakash, A. Raio, R. Riesco Jarrín, S. Tabacchioni, W.F.A. Teo and M.E. Trujillo. Apologies were received from full members H.-J. Busse, S. Dedysh, S. Emler, C.M. Manaia (vice-chair, ICSP), M. Sakamoto (full member and co-opted member of the Executive Board), M. Stott, S.N. Venter and A. Ventosa; from D.R. Arahal (chair, JC); from co-opted members P. Kuhnert, A. Nemec and J. Tambong; from J. Overmann (chair, Subcommittee on the Taxonomy of Phototrophic Bacteria); and from A. Monica (the Microbiology Society).

## Minute 4. Report of the activities of the ICSP during the current term to IUMS

At the request of R.A. Samson, Executive Secretary of the International Union of Microbiological Societies (IUMS), E.R.B. Moore submitted an addendum, covering the activities of the ICSP for the period of 1 September 2023 to 30 September 2024, to the report for the 2020–2023 term sent to R.A. Samson and the IUMS on 10 August 2023, to be presented during the IUMS Executive Board meeting to be held in Florence during the conference. The total current membership of the ICSP has grown to 56 members (35 full members, 18 co-opted members, and 3 life members).

## Minute 5. Update on co-option of new ICSP members

A. Oren explained the procedure for co-option of members to the ICSP as regulated by Article 2(b) of the statutes. The number of co-opted members was recently increased to 18, following the co-option of members from countries such as Belarus, Canada and Thailand that are not represented by an IUMS member society and others belonging to disciplines not or poorly represented in the membership, recruited from under-represented geographical areas or co-opted for the purpose of assisting with special aspects of the work of the ICSP. The co-opted members will serve until 31 March 2026.

## Minute 6. Update from ICSP judicial commission

H. Christensen reminded the attendees of the functions of the JC as defined in Article 7 of the ICSP statutes. He reported that Opinions 129 and 130 were recently issued [2,3] and that preparation of Opinion 131 in which the JC is requested to decide on the status of the prokaryotic genus name *Proteus* [4] is in progress. The JC has published guidelines for interpreting the International Code of Nomenclature of Prokaryotes (ICNP) and for the preparation of a Request for an Opinion [5].

## Minute 7. Update from the treasurer

In accordance with Article 5(c) (2) of the ICSP statutes [1], the treasurer P. Nielsen presented a statement of the accounts. Income on the GBP account is generated from royalties received from the Microbiology Society for the publication of the *IJSEM*. From this account, a stipend was paid to the Deutsche Sammlung von Mikroorganismen und Zellkulturen for maintaining the List of Prokaryotic names with Standing in Nomenclature website retroactively from 2019, as well as travel expenses for *IJSEM* Editors. As long as sufficient funds are available, *IJSEM* editors, list editors and nomenclature reviewers may claim up to 1000 £ annually as reimbursement for travel expenses to attend conferences to promote the journal. No transactions were recorded in the past year in the ICSP US$ account, which may be used to cover expenses related to the van Niel International Prize (see Minute 17).

## Minute 8. Report on Subcommittees on Taxonomy

S.L.W. On, Secretary of Subcommittees on Taxonomy, reported that currently there are 18 active subcommittees on taxonomy. The most recent addition to the list is the subcommittee on the taxonomy of Myxobacteria (Myxococcota) [6]. There has been an expression of interest in re-establishing or reactivating one or more subcommittees that ceased to be active long ago.

## Minute 9. Update on the Ad Hoc Committee on Mitigating Changes in Prokaryotic Nomenclature

M. Göker reported on the Ad Hoc Committee for Mitigating Changes in Prokaryotic Nomenclature, a committee formed in early 2024 by a group of medical and veterinary researchers and practitioners and taxonomic nomenclature experts under the auspices of the ICSP. The Ad Hoc Committee is concerned with changes in the naming of bacteria that occur in databases and publications. The increasing number of bacterial name changes has serious implications for, among other fields, medical microbiology. The committee holds bimonthly plenary meetings and bimonthly accessory meetings and started preparing lists of recommended names of prokaryotes for the medical profession. Interested colleagues are welcome to join.

## Minute 10. Update on the singleton initiative

As many type strains of species or subspecies whose names were validly published under the ICNP before 2000 are available only from a single culture collection, M. Göker initiated an effort for strains of singleton deposits to be distributed to additional culture collections to ensure the availability of type material to the research community. Several culture collections have expressed their willingness to exchange such singleton deposits, but it will take considerable time before the process is completed. In cases where type strains are no longer available from any collection, neotypes should be proposed.

## Minute 11. Update from the working group on education & outreach

S.L.W. On reported that the Working Group on Education & Outreach was relatively inactive until recently. Co-opted member M. del C. Montero-Calasanz joined the working group, and other interested colleagues are welcome to contribute.

### The ICSP website

E.R.B. Moore reported that the list of ICSP members on the ICSP website (https://www.the-icsp.org) needs updating and that minutes of the most recent meetings of the Executive Board are waiting to be uploaded.

### The ICSP on social media

M. Göker mentioned that there is little activity on the Twitter/X account of the ICSP. Suggestions on a more active use of social media to promote the activities of the ICSP are welcome.

### The usefulness of Slack as a discussion platform

A. Oren reported that in the past year, lively discussions were held on the Slack platform on proposed emendations of the ICNP and the proposed revision of the ICSP statutes. Currently, a discussion is open on a recently proposed emendation of Rule 8 of the ICNP [7]. The Slack platform is open any time for new discussions. To use the system, registration is required. Colleagues who wish to obtain access to the discussions on the Slack platform may contact A. Oren.

## Minute 12. Update from the working group on type strain accessibility

E.R.B. Moore reported that in the past, there was some difficulty obtaining type strains from some culture collections in China, but these problems have been largely resolved. A major problem has been the restriction of Chinese genetic resources by national authorities. The Chinese collections are making efforts to be able to provide strains to requests from outside of China.

A few years ago, an advisory committee was established to assist the Editor-in-Chief and the Editorial Board of the *IJSEM* on matters related to the availability of type strains and the material transfer agreements attached to them. This advisory committee, which included legal experts from two culture collections, was rarely functional. E.R.B. Moore will try to revive this committee. K.-I. Suzuki explained how the World Federation for Culture Collections (WFCC) functions and has been considering how to designate collections that are able to accept and distribute type strains. I. Kurtböke, the current President of the WFCC who was recently elected as a co-opted member of the ICSP, may be able to provide further advice.

## Minute 13. Update from the Publications Committee

E.R.B. Moore reported that the Publications Committee has not convened since the previous plenary meeting of the ICSP [8].

## Minute 14. Update from *IJSEM*

The *IJSEM* Editorial Board met in London on 6 September. The meeting was attended by ICSP Executive Board members E.R.B. Moore, C.M. Manaia, A. Oren and D.R. Arahal. The decreased impact factor of the journal was discussed. The impact factors of most microbiology journals dropped as well, and the importance of the impact factor decrease is still being analysed. Suggestions on improving the impact of the journal have been received by members of the ICSP in the past and have been discussed in the Publications Committee. New suggestions are welcome. H. Christensen asked to what extent the planned change to an open access journal might affect the future impact factor. However, it is still not clear when the *IJSEM* will become a fully open-access journal.

## Minute 15. Update from the Editor-in-Chief of the ICNP

Since the publication of the 2022 revision of the ICNP [9], many significant emendations have been approved or are expected to become approved soon. These include the addition of the ranks of Kingdom and Domain to the taxonomic hierarchy [10]; emendation of Rules 8, 15, 22, 25 a, 30(3)(b), 30(4), 34 a and Appendix 7 [11]; a proposal for further integration of *Candidatus* names into the International Code of Nomenclature of Prokaryotes, adding a new Section 10 with Rules 66–73 [12]; and emendation of Appendix 9 with new guidelines for naming genera after geographical locations [13] and for the formation of prokaryote names from personal names [14], as well as modified guidelines for the use of connecting vowels in compound names [15]. The Editorial board, consisting of A. Oren (Editor-in-Chief); ex-officio members E.R.B. Moore, C.M. Manaia, D.R. Arahal and H. Christensen; and co-opted member M. Göker, met on 14 October 2024 to prepare the 2025 revision of the ICNP. An advanced draft of the document already exists. It is the intention to finalize the draft and publish it in the *IJSEM* no later than January 2025. After a 6-month discussion period, the proposed 2025 revision of the ICNP will be submitted for balloting among the full and co-opted members of the ICSP.

## Minute 16. Update on the revision of the ICSP statutes

Some time ago, it was realized that the current version of the statutes of the ICSP [1] need updating as they sometimes lack clarity regarding important issues, contain gaps as certain aspects that should be regulated are not, may contain some unfair or unjustified regulations, do not properly implement separation of powers and more. Therefore, a revision of the statutes was initiated [16]. M. Göker explained the major changes proposed, including the option to appoint more than one Full Member per society, depending on its size, granting voting rights for Life Members, formal regulation of the work of Ad Hoc Subcommittees and easier maintenance of the Executive Board. A 6-month open discussion of the proposal, held on the Slack channel, ended on 5 September 2024. The authors of the proposed revision then adopted further revisions of the published proposals for revision. M. Göker explained the way the ballot will be presented for voting on the revised statutes. A discussion followed on the issue of the kind of majority needed for the ICSP to make decisions: a relative majority (plurality) as proposed by the authors [16] or an absolute majority as was proposed as an alternative during the discussion period. As no consensus was reached on how to present this issue in the ballot form, it was decided that the authors of the proposal should re-evaluate and, if necessary, modify the revision and the ballot form before it is sent out to the voting members.

## Minute 17. Update on IUMS 2024 Congress, Florence, 23–25 October

E.R.B. Moore reminded the audience that on 24 October, an ICSP-sponsored session would be held during the IUMS 2024 Congress (https://iums2024.com/). The session will be opened by E.R.B. Moore, chair of the ICSP. P.R. Young from the University of Queensland in Australia will give a short presentation about the van Niel International Prize and will make the introduction and presentation of the Prize Certificate to T. Woyke, the recipient of the 2020 van Niel International Prize. She will deliver her prize lecture entitled, ‘Exploring the bottom of the iceberg via single cell genomic approaches’. In addition, there will be two short lectures: a joint presentation by A. Oren and S. Ventura on the history of the ICSP and a presentation by M. Göker on the recent activities of the ICSP.

E.R.B. Moore reminded the audience that, although the ICSP nominates the van Niel International Prize awardee to the University of Queensland, the ICSP does not have any financial obligations with respect to the prize. The ICSP has agreed to cover the travel and accommodation costs of T. Woyke in this instance but should not be considered to be a precedent. E.R.B. Moore will further discuss the financial arrangements for future awardees of the van Niel Prize with P.R. Young and officials of the University of Queensland.

## Minute 18. Open session for attendees to present questions and discussion points

No questions or discussion topics were brought forward by the attendees.

### Closing matters

E.R.B. Moore announced his intention to prepare an online survey in which the ICSP members will be asked about the items of this plenary meeting and what they would like to discuss during future plenary meetings.

### Date/time of the next plenary meeting

The next plenary meeting, to be held online, was tentatively scheduled to take place in May 2025, preferably at a time that is convenient for delegates in the Far East, India, Australia and New Zealand.

The meeting was adjourned at 17.15 CEST.

## Abbreviations

ICNP: International Code of Nomenclature of Prokaryotes
ICSP: International Committee on Systematics of Prokaryotes
*IJSEM*: *International Journal of Systematic and Evolutionary Microbiology*
IUMS: International Union of Microbiological Societies
JC: Judicial Commission
WFCC: Word Federation for Culture Collections
